# Clinical benefit of oral lactulose for postoperative care of pateints with complicated appendicitis using propensity score matching analysis

**DOI:** 10.1186/s12876-019-1073-2

**Published:** 2019-08-30

**Authors:** Xin Ding, Jiaming Lan, Bailin Chen, Lin Qiu, Chunbao Guo

**Affiliations:** 1 Department of Pediatric General Surgery and Liver Transplantation, Children’s Hospital, Chongqing Medical University, Chongqing, 136 Zhongshan 2nd Rd, Chongqing, 400014 People’s Republic of China; 2 Department of Burns and Plastic Surgery, Children’s Hospital, Chongqing Medical University, Chongqing, People’s Republic of China; 3 Ministry of Education Key Laboratory of Child Development and Disorders, Children’ss Hospital, Chongqing Medical University, Chongqing, People’s Republic of China; 4 Key Laboratory of Pediatrics in Chongqing (CSTC2009CA5002), Chongqing International Science and Technology Cooperation Center for Child Development and Disorders, Chongqing, China

**Keywords:** Lactulose, Gastrointestinal function, Postoperative complications, Adhesive small bowel obstruction

## Abstract

**Objective:**

The aim of this study was to investigate the effect of oral lactulose for pediatric patients with complicated appendicitis, who underwent appendectomy.

**Background:**

Oral lactulose was widely used for gastrointestinal function regulation. However, clinical benefit for oral lactulose regarding its effects on recent postoperative gastrointestinal (GI) recovery and long term adhesive small bowel obstruction (ASBO) incidence, especially in the postoperative pediatric population has not yet defined.

**Methods:**

A total of 525 pediatric patients with complicated appendicitis underwent appendectomy were retrospectively reviewed. Among them, 317 cases were subjected to oral lactulose management and 208 patients without, served as control. Propensity score 1:1 matching was carried out to adjust for any potential baseline variables. In 189 paired patients, clinical outcomes, including gastrointestinal recovery variables, incidence of ABSO, as well as adverse events, were compared according to the oral lactulose administration or not.

**Results:**

Patients who received oral lactulose administration achieved early gastrointestinal function recovery, including, first bowel movement (Risk ratio [RR], 1.34; 95% confidence interval [CI] 1.02–2.63, *p* = 0.005) and first solid feeding (RR, 1.26; 95% CI, 1.01–1.92, *p* = 0.012). A lower occurrence of ASBO (OR, 0.47; 95% CI, 0.25–0.87; *p* = 0.011) and lower constipation (Odds ratio [OR], 0.25; 95% CI, 0.13–0.46; *p* < 0.001), were noted in patients received oral lactulose than in patients without. Furthermore, significantly fewer patients required readmission (OR, 0.56; 95% CI, 0.32–0.99; *p* = 0.031) and reoperation (OR, 0.29; 95% CI, 0.09–0.92; *p* = 0.022) in the patients who received oral lactulose administration.

**Conclusions:**

Beneficial effects of oral lactulose administration in pediatric patients undergone appendectomy were indicated, such as accelerating gastrointestinal function recovery, reducing the postoperative incidence of ASBO and constipation, so reduced readmission and reoperation.

## Background

Complicated appendicitis (periappendiceal abscess or pan-peritonitis) was associated with severe inflammatory secretion, which is the main etiologic factor for postoperative ileus (POI), and which developed with pain, nausea, vomiting, distension and the inability to take sufficient liquids and solids [1, 2]. Accelerating gastrointestinal recovery could increase patient comfort, reduce surgical complications, decrease the average hospital length of stay (LOS), and other demands on healthcare resources [3].

Core accelerated care pathways included no perioperative fasting, optimal nutrition and fluid management, decreased use of tubes, optimizing pain control, and early mobilization have been used in some specialized centers to facilitate gastrointestinal (GI) recovery [4, 5]. Furthermore, postoperative adhesive small bowel obstruction (ASBO), is the most common surgical causes for hospital readmission, bowel resection [6], but until now, no specific treatment currently exists for the treatment or prevention of POI and ASBO. Alvimopan has been suggested with possible therapeutic effect in accelerated the recovery of bowel function [7]. Sesame oil and Gastrografin has been used for postoperative ASBO though relieve constipation and keep bowel movements regular. The advantage of this treatment was effective, safe and no radiation involvement [8, 9]. The laxatives confers therapeutic benefits by guiding water into the intestinal lumen, promoting smooth muscle contractility and decreasing the intestinal edema [10]. Information for oral lactulose regarding its effects on postoperative care in pediatric populations is sparse.

In our institute, oral lactulose was prescribed in some patients according to the preference and experience of some of surgical team. We retrospectively reviewed a consecutive series of pediatric patients following appendectomy to compare the therapeutic role of oral lactulose, in terms of intestinal function recovery, and ASBO incidence.

## Methods

### Patients

The retrospective review was conducted with the patients underwent appendectomy for complicated appendicitis in a general surgery department of a tertiary hospital from Aug. 2013 to Aug. 2018. The hospital ethics committee for medical research of the Children’s Hospital of Chongqing Medical University approved the study protocol. Consent was obtained from a parent or guardian on behalf of any participants under the age of 16. Pediatric patients undergoing emergency appendectomy, either laparoscolopic or open approach, were eligible for entry into the study upon meeting the following inclusion criteria: patients age > 1 year and < 16 years; evidence of complicated appendicitis; normal renal and hepatic function. Exclusion criteria included evidence of simple appendicitis; poor compliance of lactulose administration; previous abdominal operation; interval appendectomy; narcotic utilization for pain management; incomplete follow-up data, etc.

### Managements

In our institution, complicated appendicitis was confirmed during the emergency surgical interventions, including laparoscopic or open procedures. Following the emergency surgery, all patients underwent the same postoperative programme, including enteral feeding, early ambulation and fluid resuscitation if necessary. The accelerated postoperative care protocol was used to promote the recovery of GI function. On postoperative day 1, ambulation was encouraged; liquids and solid food was offered in proper order from postoperative day 1 or day 2. If kept in place after surgery, the nasogastric tube (NGT) was removed within 24 h after surgery. Probiotics, apocrustic agents or atropine, when appropriate were symptomaticly used for diarrhea and abdominal cramping or bloating. Other adverse symptoms were managed as indicated clinically. Postoperatively, all the patients were administered intravenous broad-spectrum antibiotics continuously with either piperacillin/tazobactam (100–150 mg/kg/day in two divided doses) or cefoperazone sodium/sulbactam (100–150 mg/kg/dose in two divided doses) and metronidazole (30 mg/kg/dose in two divided doses) for about 5 days until the serum C-reactive protein (CRP) concentrations decreased to below 1.0 mg/dL. Choice of lactulose depended on the patient condition and surgeon’s preference, because some surgeons in our institution felt that it might be necessary and safe for lactulose administration. The lactulose was started on the first day after surgery in some patients once daily and continued for a maximum of 5 years to stimulate bowel function, if a patient did not spontaneously defaecate with 2 days. The dose of lactulose was adjusted to the target of defecate once a day.

### Clinical data and outcome evaluation

Following institutional review board approval, the medical records of all included patients were thoroughly reviewed by two independent investigators who had undergone our specific training. The collected data available in the medical records included the patients’ clinical history and physical examination findings on admission, like onset and course of nausea/vomiting, crampy pain, distension, fever, bowel sounds; laboratory data on admission, including venous blood leukocyte count, CRP if available and serum electrolytes prior to the oral lactulose; the original imaging examination on admission, including B ultrasound, or computed tomography (CT) scans; operative data, like surgical approach (conventional or laparoscopic), as well as the postoperative outcomes, including Ggastrointestinal symptoms (first bowel movement [gas and feces] after operation, abdominal bloating, abdominal cramps, diarrhea [defined as more than three bowel movements per day], and vomiting), intra-operative microbiological data, infectious complications (wound infection or granulom, peritonitis or abscess). Obstruction event was defined as nausea, vomiting or abdominal pain lasting less than 3 days after the operation. Prolonged ileus was defined as a sustained non-mechanical obstruction lasting more than 5 days after the operation and confirmed by simple abdominal radiography.

### Follow up

In our institute, all patients were systematically followed up through telephone call at 6, 12 and 24 months by the investigators. Some patients were followed at the outpatient clinic at which time the questionnaire for postoperative symptoms were filled out. The date and type of obstruction event managed in the same or in another surgical unit were also collected. Twenty-seven patients (9%) were lost to follow-up and two third over 2 years of follow-up. The ASBO readmission were defined as the hospital admission for presence of abdominal pain and distention, vomiting, and complete constipation (gas and feces), which were confirmed by the abdominal film.

The primary outcome measure was the incidence of readmission for ASBO. the time span of readmission was measured from the day of the primary operation to the readmission. The acute adhesive small bowel obstruction (ASBO) episode was defined as an admission to hospital for a patient already undergone abdominal surgery in the past, with the following diagnostic criteria: presence of abdominal pain, vomiting, complete constipation (gas and feces), or the attending surgeon giving the diagnosis of acute ASBO. Secondary outcomes included the GI function recovery variables, including first bowel movement, postoperative solid feeding time, the perioperative complications, and hospital LOS (the total number of days from the admission to discharge), etc.

### Statistical analysis

As potential confounders could affect the readmission for obstruction and lactulose was not used in a randomized manner in our study, we established propensity score matching using SPSS 20.0 (IBM, Armonk, NY) or R 3.1.2 (The R Foundation for Statistical Computing) to avoid the potential confounders. A 1:1 Propensity scores matching was performed using the nearest neighbor without replacement matching algorithm and a 0.1 caliper width. The generalized additive model was used to check linear assumption in PS model. After PS matching, statistical comparisons were conducted between the matched lactulose treatment patients and controls using SPSS 20.0 (IBM, Armonk, NY). Continuous data were expressed as means ± SDs or median (interquartile range [IQR]) and were analyzed using Student’s t-test or Mann-Whitney U test respectively. The discrete variables was analyzed by a chi-square test or Fisher’s exact test, and then by estimation of the relative risk between treatment groups. The relative risks for postoperative variables were assessed using cross-tabulation (odds ratio [OR]) or multivariate logistic regression analysis (risk ratio [RR]). The statistical significance was evaluated using a two-tailed 95% confidence interval (CI), and statistical significance was established if *P* < 0.05.

## Results

### Patient characteristics

Among the initial 525 pediatric patients eligible for analysis, 317 (60.4%) received postoperative laxative administration. The baseline features of the patients according to laxative administration are summarized in Table 1. Before PS-matching, there were no significant differences in terms of age, sex, laboratory test (like, white blood cell counts, CRP concentrations), duration of symptoms on admission between the two groups, suggesting that there were no systematic differences in baseline characteristics between the two groups. Under PS-matching, 189 patients with laxative administration were matched to 189 patients without. There were no significant differences in surgical approach between the two groups with unmatched and propensity score matched patients (Table 1). Several variables, including operation type, became more comparable after PS-matching (Table 1). Under PS-matching, the absolute standardized mean differences reduced the values to the range from 0.01 to 0.07, indicating that the continuous and categorical variables were very similar and comparable between the patients with and without laxative administration (Table 1).

**Table 1:** Baseline demographicsand preoperative variables of eligible population

	Total population			Propensity matched population		
Lactulose	With (317)	Without (208)	*P* Values	With (189)	Without (189)	*P* Values
Age (yrs), Mean ± SD	6.2 ± 1.8	6.1 ± 1.7	0.19	6.1 ± 1.7	6.1 ± 1.8	0.33
Male: female	186:131	120:88	0.45	108:81	109:80	0.50
Mean body weight (kg), Mean ± SD	13.9 ± 5.7	13.8 ± 5.8	0.36	13.8 ± 5.4	13.8 ± 5.2	0.62
WBC (10^9^/L), Mean ± SD	17.5 ± 4.9	18.2 ± 5.2	0.24	17.6 ± 4.5	17.9 ± 4.9	0.39
PCT (ng/ml, normal value: 0–0.5)	6.7 ± 2.1	6.9 ± 2.2	0.33	6.8 ± 2.0	6.8 ± 2.1	0.52
CPR (mg/L, normal value: 0–10)	14.1 ± 4.9	13.9 ± 4.7	0.18	14.0 ± 4.2	13.9 ± 4.3	0.36
Duration of symptoms on admission (h), days, Mean ± SD	2.7 ± 1.4	2.9 ± 1.6	0.26	2.8 ± 1.5	2.8 ± 1.6	0.38
Clinical symptoms, N (%)						
Abdominal pain	294 (92.7)	193 (92.8)	0.57	175 (92.6)	175 (92.6)	0.58
Vomiting	183 (57.7)	122 (58.7)	0.45	109 (57.7)	110 (58.2)	0.50
Fever	152 (47.9)	98 (47.1)	0.46	90 (47.6)	90 (47.6)	0.50
Appendicolith	113 (35.6)	66 (31.7)	0.20	64 (33.9)	62 (32.8)	0.46
pan-peritonitis	82 (25.9)	57 (27.4)	0.39	50 (26.5)	51 (27.0)	0.50
Abscess 2 cm or more, N (%)	63 (19.9)	42 (20.2)	0.51	38 (20.1)	38 (20.1)	0.55
Operation type, N (%)						
Laparoscopical	193 (60.9)	127 (61.1)	0.52	115 (60.8)	116 (61.4)	0.50
Open	124 (29.1)	81 (28.9)		74 (29.2)	73 (28.4)	

Abbreviation: *WBC* white blood cell, *PCT* procalcitonin, *CRP* C-reactive protein

### Efficacy for gastrointestinal function recovery

A positive trend for accelerated GI function recovery was observed for patients treated with laxative, which was assessed by first bowel movement, first stool, and postoperative feeding time. The first bowel movements occurred 1.6 ± 0.6 days and 2.2 ± 0.8 days after surgery in the patients with and without laxative treatment, respectively (Risk ratio [RR], 1.34; 95% confidence interval [CI] 1.02–2.63, *p* = 0.005). The first solid feeding is faster in the patients with laxative treatment than without (RR, 1.26; 95% CI, 1.01–1.92, *p* = 0.012), whereas no difference was found for first flatus (*p* = 0.082). In the successful laxative treated cases, 77.8% (137/189) patients with oral laxative treatment spontaneously passed stool within 72 h, whereas only 47.1% (121/189) cases in the control group passed stool within the same duration (RR, 1.48; 95%CI 0.96–2.29, *p* = 0.049). There were no differences in the incidence of diarrhea or serum electrolyte abnormalities between the two groups. The mean length of hospital stay was 8.9 ± 1.7 days in patients receiving laxative, which was significantly less than the mean length of stay (9.7 ± 1.6 days) in patients without laxative treatment (RR, 0.54; 95% CI, 0.32–0.98, *p* = 0.038) (Table 2).

**Table 2:** Gastrointestinal function and early outcome in the matched population

Lactulose	With (189)	Without (189)	*P* values	Risk ratio (95% CI)
First flatus, days, Mean ± SD	2.1 ± 0.9	2.5 ± 1.1	0.082	1.19 (0.83–1.69)
first bowel movement, Mean ± SD	1.6 ± 0.6	2.2 ± 0.8	0.005	1.34 (1.02–2.63)
passed stool within 72 h, N (%)	137 (72.5)	121 (64.0)	0.049	1.48 (0.96–2.29)
Postoperative NGT, N (%)	28 (14.8)	39 (20.6)	0.089	0.67 (0.39–1.14)
x-ray measurement, N (%)	36 (19.0)	50 (26.5)	0.055	0.65 (0.40–1.06)
First solid feeding, days, Mean ± SD	2.0 ± 0.4	2.6 ± 0.5	0.012	1.26 (1.01–1.92)
Obstruction event, N (%)	71 (37.6)	57 (30.2)	0.13	1.39 (0.91–2.14)
Abdominal distension after POD 5, N (%)	39 (20.6)	47 (24.9)	0.20	0.79 (0.49–1.27)
CRP at POD 5, mg/L, Mean ± SD	22.2 ± 6.7	26.6 ± 7.3	0.053	0.72 (0.53–1.97)
Postoperative hospital stay, days, Mean ± SD	8.9 ± 1.7	9.7 ± 1.6	0.038	0.54 (0.32–0.98)

Abbreviation: *NGT* nasogastric tube, *POD* postoperative day

### Follow-up and recurrence rates

The median follow-up was 31 months (range, 12–89 months). During follow-up, the incidence of constipation in the patients with oral laxative administration was significant lower compared with that in the control (OR, 0.25; 95% CI, 0.13–0.46; *p* < 0.001), which was expected. The most common reasons for the hospital readmission were surgery related ABSO. Significant less incidence of ASBO was observed in the patients who received laxative (OR, 0.47; 95% CI, 0.25–0.87; *p* = 0.011) compared with the control (Table 3). Among them, the median time of first recurrent ASBO was 3.6 months for laxative group and 2.8 months for control after operation. Of those patients who received laxative, 22 cases (11.6%) of patients within 24 months were readmitted to the hospital for ABSO and 36 cases (18.5%) of patients in the control, suggesting that laxative reduced the need for hospital readmission (OR, 0.56; 95% CI, 0.32–0.99; *p* = 0.031). Of the 17 patients who required surgical intervention, four patients (2.1%) with laxative were subjected with emergency surgery and 11 (5.8%) control patients had re-operation (OR, 0.29; 95% CI, 0.09–0.92; *p* = 0.022) for suspected bowel strangulation (Table 3). The bowel strangulation segments were underwent resection in 2 cases in laxative group, and 6 cases in the control.

**Table 3:** Follow-up and recurrence rates in the matched population

Lactulose	With (189)	Without (189)	*P* Values	Odds ratio (95% CI)
constipation, N (%)	15 (7.9)	49 (25.9)	< 0.001	0.25 (0.13–0.46)
incidence of prolonged POI, N (%)	28 (14.8)	37 (19.6)	0.14	0.71 (0.42–1.22)
incidence of ASBO	17 (9.0)	33 (17.5)	0.011	0.47 (0.25–0.87)
time of first recurrent ASBO, month, median (range)	3.6 (2.5–12)	2.8 (1.7–15)	0.12	
Hospital readmission, N (%)	22 (11.6)	36 (18.5)	0.031	0.56 (0.32–0.99)
re-operation for ASBO, N (%)	4 (2.1)	13 (5.8)	0.022	0.29 (0.09–0.92)
Bowel resection	2 (1.1)	6 (3.2)	0.14	

Abbreviation: *POI* postoperative ileus, *ASBO* adhesive small bowel obstruction

### Safety assessments

In general, oral laxative was well tolerated with similar safety profiles among oral laxative administration and control groups. The patients using laxative suffered almost equal early postoperative complications in terms of anastomotic leakage, incision dehiscence, wound infection, intraperitoneal abscess, etc. Postoperative total adverse events (AEs), like nausea or vomiting, abdominal cramps, and abdominal distention in the two groups were comparable (Table 4). Nausea and vomiting were the most common AEs that led to study discontinuation. About 18 patients (9.5%) should shortly discontinue the laxative administration by the nausea and vomiting. Furthermore, there was no difference in the incidence of diarrhea and serum electrolyte abnormalities between the two groups.

**Table 4:** Postoperative complication sand treatment-emergent adverse events in the matched population

Lactulose	With (189)	Without (189)	*P* Values	Risk ratio (95% CI)
Total complications (at least 1 complication), N (%)	44 (23.3)	39 (20.6)	0.31	1.17 (0.72–1.90)
Anastomotic leakage, N (%)	2 (1.1)	1 (0.5)	0.50	2.01 (0.18–22.36)
Incision dehiscence, N (%)	6 (3.2)	7 (3.7)	0.78	0.85 (0.28–2.59)
Peritonitis or abscess, N (%)	26 (13.8)	31 (16.4)	0.28	0.81 (0.46–1.43)
Total AEs, N (%)	75 (39.7)	86 (45.5)	0.25	0.79 (0.52–1.19)
Nausea or Vomiting, N (%)	35 (18.5)	31 (16.4)	0.59	1.16 (0.68–1.97)
Abdominal cramps, N (%)	27 (14.3)	25 (13.2)	0.44	1.09 (0.61–1.96)
Diarrhea, N (%)	33 (17.5)	26 (13.8)	0.32	1.33 (0.76–2.32)
Serum electrolyte abnormalities, N (%)	15 (7.9)	18 (9.5)	0.36	0.82 (0.40–1.68)

Abbreviation: *AEs* adverse events

## Discussion

After propensity score matching of heterogeneity in the population, the current analysis verified that oral laxative significantly accelerated GI function (bowel movements) recovery in patients undergoing appendectomy. Laxative also significantly accelerated the toleration of oral feed, which is important for hospital discharge. Furthermore, patients with the laxative administration experienced low incidence of readmission and reoperation because of the ASBO, which is a clinically important advert event for the current surgery.

Postoperative intestinal recovery serves as the main focus of abdominal surgery [11, 12]. Postoperative ileus detention might lead to oral nutrition delay, prolonged hospital stay, even with hospital readmission [13]. Various protocols, including minimally invasive surgical intervention (eg, laparoscopy instead of laparotomy), early oral nutrition, and physical rehabilitation have been suggested to accelerate postoperative GI recovery [14, 15]. Laxatives have also been studied with significantly earlier bowel function recovery in patients undergoing hysterectomy in a randomized study [10]. The presence of laxative could promotes shifting of fluid into the intestinal lumen, which might stimulate bowel activity for proper intestinal function recovery [16]. The current analysis confirmed that laxative significantly increased the proportion of patients who achieved GI function recovery during the early recovery periods in the pediatric patients with complicated appendicitis who underwent appendectomy. Our analysis involves laxative usage in a closely monitored hospital setting, with frequent nursing assessments and continuous intestinal function monitoring. Therefore, the clinically significant intestinal complaints would likely be captured. We found that laxative usage was associated with shifting more patients into the earlier phase of the recovery process. In out institute, an accelerated care protocol was encouraged to facilitate GI recovery, including early oral nutrition and ambulation in patients undergoing major intestinal surgery. This practice not only accelerated GI recovery but resulted in shorter LOS compared with traditional care. However, the length of hospital stay for the laxative group in this analysis was indeed less than the balanced control. Therefore, the benefits of laxative were beyond those provided by accelerated postoperative care pathways alone.

According to previous reports, readmission within 30 days was required for about 3.7% of patients following the appendectomy and 7.7% (666/8688) for patients with perforated appendicitis [17]. Under our management protocol, the overall need for readmission was lower in the laxative administration patients compared with the control. Accordingly, the current laxative administration reduces the need for surgical intervention in adhesive small bowel obstruction, which has not been reported in the literature before. Previous report suggested that ranged from 6 to 11% of ASBO might develop to bowel strangulation [18, 19]. In the current study, the overall strangulation rate was only 0.8% for patients with laxative administration. We believe that beside its osmotic nature, other mechanisms may be involved in the current performance of laxative. Laxative may also reduce the inflammatory response of postoperative gastrointestinal tract, which was characteristic by edema and inflammation [20, 21], and thereby reduce ASBO. In the current research, CRP enhanced quickly following the operation and recovered better with laxative administration compared with the control, confirm the dampening of local inflammation by laxative, which might explain the current clinical results, although the exact mechanism is difficult to determine in this clinical setting.

In theory, stimulation of the bowel might cause bowel strangulation or anastomotic dehiscence [22]. But in the present study, there was no complication or mortality that could be attributed to the laxative usage. The incidence of bowel strangulation or anastomotic leak was comparable for the two groups, supporting the point that promoting GI recovery by laxative should not compromise with postoperative complications. There was also no evidence that the use of laxative would increase the risk of nausea, abdominal cramps. Because of the fluid shifting into bowel lumen, it may further dehydrate the patient following the adverse effect of obstruction [23, 24]. The oral laxative is very little absorption making this drug quite safe to use even for a long time. In fact, adverse effects for laxative usage in small bowel obstruction have rarely been mentioned, including serum electrolyte abnormalities and other adverse events. Furthermore it is important for controlled diet and the reabilitation treatment for the canalization, which should be preceded by re-educative treatment [25].

There are several limitations to our study, while it is the largest reported series of patient with oral laxative usage following major gastrointestinal surgery. First, this is a single-center retrospective study. The decision to initiate laxative was not made randomly with inherent risk of selection bias. The GI-associated intestinal function, like the days to defecation, first flatus and cramps were extracted from clinical records of patient, which should not be fully objective and accurate. We could not completely avoid variables that may have affected our results as residual confounders. Therefore, our results should be interpreted cautiously. Another point is that the patients with laxative administration might be more surgically challenged than the control, with more potential SBO. To avoid this, we performed propensity score matching analysis to generate comparison confounding variables on the actual effects of laxative.

## Conclusion

The present data suggested that oral laxative accelerated GI recovery and reduced postoperative morbidity and readmission after appendectomy in the pediatric patients with complicated appendicitis. We acknowledge that these results are based on retrospective review of the homogenous group of patients. Thus, further trials with large patient samples randomized controlled clinical trials are required to confirm these effects.

## Data Availability

The datasets during and/or analyzed during the current study are available from the corresponding author on reasonable request.

## Abbreviations

AEs: Adverse events
ASBO: Adhesive small bowel obstruction
CI: Confidence interval
CRP: C-reactive protein
CT: Computed tomography
GI: Gastrointestinal
IQR: Interquartile range
LOS: Hospital length of stay
NGT: Nasogastric tube
OR: Odds ratio
POI: Postoperative ileus
RR: Risk ratio

## References

[1] Horvath P, Lange J, Bachmann R, Struller F, Königsrainer A, Zdichavsky M (2017). Comparison of clinical outcome of laparoscopic versus open appendectomy for complicated appendicitis. Surg Endosc.

[2] Schlottmann F, Reino R, Sadava EE, Campos Arbulú A, Rotholtz NA (2016). Could an abdominal drainage be avoided in complicated acute appendicitis? Lessons learned after 1300 laparoscopic appendectomies. Int J Surg.

[3] ERAS Compliance Group (2015). The impact of enhanced recovery protocol compliance on elective colorectal cancer resection: results from an international registry. Ann Surg.

[4] Liu VX, Rosas E, Hwang J, Cain E, Foss-Durant A, Clopp M, Huang M, Lee DC, Mustille A, Kipnis P, Parodi S (2017). Enhanced recovery after surgery program implementation in 2 surgical populations in an integrated health care delivery system. JAMA Surg.

[5] Ahmed J, Khan S, Lim M, Chandrasekaran TV, MacFie J (2012). Enhanced recovery after surgery protocols - compliance and variations in practice during routine colorectal surgery. Color Dis.

[6] Mu JF, Wang Q, Wang SD, Wang C, Song JX, Jiang J, Cao XY (2018). Clinical factors associated with intestinal strangulating obstruction and recurrence in adhesive small bowel obstruction: a retrospective study of 288 cases. Medicine (Baltimore).

[7] Harms BA, Heise CP (2007). Pharmacologic management of postoperative ileus: the next chapter in GI surgery. Ann Surg.

[8] Lee CY, Hung MH, Lin LH, Chen DF (2015). Evaluation of a water-soluble contrast agent for the conservative management of adhesive small bowel obstruction in pediatric patients. J Pediatr Surg.

[9] Ji ZL, Li JS, Yuan CW, Chen WD, Zhang YN, Ju XT, Tang WH (2010). Therapeutic value of sesame oil in the treatment of adhesive small bowel obstruction. Am J Surg.

[10] Hansen CT, Sørensen M, Møller C, Ottesen B, Kehlet H (2007). Effect of laxatives on gastrointestinal functional recovery in fast-track hysterectomy: a double-blind, placebo-controlled randomized study. Am J Obstet Gynecol.

[11] Stakenborg Nathalie, Labeeuw Evelien, Gomez-Pinilla Pedro J, De Schepper Sebastiaan, Aerts Raymond, Goverse Gera, Farro Giovanna, Appeltans Iris, Meroni Elisa, Stakenborg Michelle, Viola Maria Francesca, Gonzalez-Dominguez Erika, Bosmans Goele, Alpizar Yeranddy A, Wolthuis Albert, D’Hoore Andre, Van Beek Kim, Verheijden Simon, Verhaegen Marleen, Derua Rita, Waelkens Etienne, Moretti Milena, Gotti Cecilia, Augustijns Patrick, Talavera Karel, Vanden Berghe Pieter, Matteoli Gianluca, Boeckxstaens Guy E (2018). Preoperative administration of the 5-HT4 receptor agonist prucalopride reduces intestinal inflammation and shortens postoperative ileus via cholinergic enteric neurons. Gut.

[12] Shang Y, Guo C, Zhang D (2018). Modified enhanced recovery after surgery protocols are beneficial for postoperative recovery for patients undergoing emergency surgery for obstructive colorectal cancer: a propensity score matching analysis. Medicine (Baltimore).

[13] Tevis SE, Carchman EH, Foley EF, Harms BA, Heise CP, Kennedy GD (2015). Postoperative ileus--more than just prolonged length of stay?. J Gastrointest Surg.

[14] Lee L, Li C, Landry T, Latimer E, Carli F, Fried GM, Feldman LS (2014). A systematic review of economic evaluations of enhanced recovery pathways for colorectal surgery. Ann Surg.

[15] Zhuang CL, Ye XZ, Zhang XD, Chen BC, Yu Z (2013). Enhanced recovery after surgery programs versus traditional care for colorectal surgery: a meta-analysis of randomized controlled trials. Dis Colon Rectum.

[16] Gonzalez-Martinez MA, Ortiz-Olvera NX, Mendez-Navarro J (2014). Novel pharmacological therapies for management of chronic constipation. J Clin Gastroenterol.

[17] Moghadamyeghaneh Z, Hwang G, Hanna MH, Carmichael JC, Mills S, Pigazzi A, Stamos MJ (2016). Unplanned readmission after appendectomy. Am J Surg.

[18] Teixeira PG, Karamanos E, Talving P, Inaba K, Lam L, Demetriades D (2013). Early operation is associated with a survival benefit for patients with adhesive bowel obstruction. Ann Surg.

[19] Bilderback PA, Massman JD, Smith RK, La Selva D, Helton WS (2015). Small bowel obstruction is a surgical disease: patients with adhesive small bowel obstruction requiring operation have more cost-effective care when admitted to a surgical service. J Am Coll Surg.

[20] Hao F, Guo H, Zhong J, Geng Q, Yang Y, Chen B, Guo C (2018). Effects of prostaglandin E1 on patients undergoing major gastrointestinal surgery. Ann Surg.

[21] Fan Y, Jiang Y, Fu X, Cai J, Wang Y, Li W, Gu X, Xing K, Bai S, Bi X (2015). Effects of liposomal prostaglandin E1 on periprocedural myocardial injury in patients with unstable angina undergoing an elective percutaneous coronary intervention. Coron Artery Dis.

[22] Vester-Andersen M, Lundstrøm LH, Møller MH, Waldau T, Rosenberg J, Moller AM (2014). Danish Anaesthesia database. Mortality and postoperative care pathways after emergency gastrointestinal surgery in 2904 patients: a population-based cohort study. Br J Anaesth.

[23] Thomas RH, Luthin DR (2015). Current and emerging treatments for irritable bowel syndrome with constipation and chronic idiopathic constipation: focus on prosecretory agents. Pharmacotherapy..

[24] Brusciano L, Limongelli P, del Genio G, Sansone S, Rossetti G, Maffettone V, Napoletano V, Sagnelli C, Amoroso A, Russo G, Pizza F, Del Genio A (2007). Useful parameters helping proctologists to identify patients with defaecatory disorders that may be treated with pelvic floor rehabilitation. Tech Coloproctol.

[25] Brusciano L., Gambardella C., Tolone S., del Genio G., Terracciano G., Gualtieri G., Schiano di Visconte M., Docimo L. (2019). An imaginary cuboid: chest, abdomen, vertebral column and perineum, different parts of the same whole in the harmonic functioning of the pelvic floor. Techniques in Coloproctology.

